# Mono-PEGylation of a Thermostable Arginine-Depleting Enzyme for the Treatment of Lung Cancer

**DOI:** 10.3390/ijms21124234

**Published:** 2020-06-14

**Authors:** Sai-Fung Chung, Chi-Fai Kim, Sui-Yi Kwok, Suet-Ying Tam, Yu Wai Chen, Hiu-Chi Chong, Siu-Lun Leung, Pui-Kin So, Kwok-Yin Wong, Yun-Chung Leung, Wai-Hung Lo

**Affiliations:** Department of Applied Biology and Chemical Technology, Lo Ka Chung Research Centre for Natural Anti-Cancer Drug Development and State Key Laboratory of Chemical Biology and Drug Discovery, The Hong Kong Polytechnic University, Hung Hom, Kowloon, Hong Kong, China; 16900402r@connect.polyu.hk (S.-F.C.); stephen.kim@polyu.edu.hk (C.-F.K.); christine.kwok@newbinnovation.com (S.-Y.K.); sabrinasy.tam@connect.polyu.hk (S.-Y.T.); yu-wai.chen@polyu.edu.hk (Y.W.C.); steve.h.c.chong@gmail.com (H.-C.C.); alan.sl.leung@polyu.edu.hk (S.-L.L.); pui-kin.so@polyu.edu.hk (P.-K.S.)

**Keywords:** L-Arg, mono-PEGylation, thermostable enzyme

## Abstract

L-arginine (L-Arg) depletion induced by randomly PEGylated arginine deiminase (ADI-PEG20) can treat arginosuccinate synthase (ASS)-negative cancers, and ADI-PEG20 is undergoing phase III clinical trials. Unfortunately, ASS-positive cancers are resistant to ADI-PEG20. Moreover, the yield of ADI production is low because of the formation of inclusion bodies. Here, we report a thermostable arginine-depleting enzyme, *Bacillus caldovelox* arginase mutant (BCA-M: Ser^161^->Cys^161^). An abundant amount of BCA-M was easily obtained via high cell-density fermentation and heat treatment purification. Subsequently, we prepared BCA-M-PEG20, by conjugating a single 20 kDa PEG monomer onto the Cys^161^ residue via thio-chemistry. Unlike ADI-PEG20, BCA-M-PEG20 significantly inhibited ASS-positive lung cancer cell growth. Pharmacodynamic studies showed that a single intraperitoneal injection (i.p). administration of 250 U/mouse of BCA-M-PEG20 induced low L-Arg level over 168 h. The mono-PEGylation of BCA-M prolonged its elimination half-life from 6.4 to 91.4 h (a 14-fold increase). In an A549 lung cancer xenograft model, a weekly administration of 250 U/mouse of BCA-M-PEG20 suppressed tumor growth significantly. We also observed that BCA-M-PEG20 did not cause any significant safety issue in mouse models. Overall, BCA-M-PEG20 showed excellent results in drug production, potency, and stability. Thereby, it has great potential to become a promising candidate for lung cancer therapy.

## 1. Introduction

Lung carcinoma is one of the top-ranking killer cancers worldwide, with poor survival rates. There are two main types—non-small cell lung cancer (NSCLC) and small cell lung cancer (SCLC); together they account for 80–85% and 10–15% of all lung cancer cases, respectively [1,2]. Conventional treatment options for lung cancer, surgery, radiotherapy, and chemotherapy, are far from satisfactory in both aspects of efficacy and adverse side effects. Thus, alternative strategies are urgently required for the treatment of lung cancer [3]. L-arginine (L-Arg) depletion is a new, promising, and safe strategy to treat several cancer types, including, leukemia, melanoma, colorectal cancer, brain cancer, and hepatocellular carcinoma. They have been reported to be arginine-auxotrophic cancers because these cells do not express argininosuccinate synthetase (ASS) or ornithine transcarbamylase (OTC) in the urea cycle [4,5,6,7,8]. Thereby, arginine-depleting enzymes may be an effective and safe way of treating lung cancers.

Arginine deiminase (ADI) is a bacterial homodimeric enzyme that shows a high catalytic rate of converting L-Arg into L-citrulline under physiological conditions [9]. Native ADI is immunogenic and is eliminated rapidly from the circulation [10]. However, random and multiple 20 kDa polyethylene glycol (PEG) covalent conjugation of ADI (ADI-PEG20) dramatically reduced the immunogenicity and prolonged the circulation half-life. This agent is undergoing phase II and III clinical trials [10]. Recently, it was reported that seven out of nine patients with malignant pleural mesothelioma and NSCLC who received cocktail therapy (ADI-PEG20, pemetrexed, and cisplatin) achieved a partial response [11]. Although ADI-PEG20 is a promising anticancer drug, its production cost is a big concern. It has been reported that recombinant ADI expressed in *E. coli* aggregated into inclusion bodies during induction [12,13,14], which necessitated complicated and extensive processing involving unfolding and refolding to produce the bioactive protein [15]. Additionally, with random PEGylation, it is difficult to produce a homogeneous product, resulting in batch to batch variations [16]. In addition, the 20 kDa succinimidyl succinate PEG used in the random PEGylation [5] can be easily hydrolyzed [17]. On top of all these engineering issues, many cancer cells are argininosuccinate synthase (ASS)-positive but ornithine transcarbamylase (OTC)-negative [8], meaning that they are intrinsically resistant to ADI-PEG20 [5,7,8,11,18,19,20,21,22]. Apart from ADI-PEG20, another PEGylated arginine-depleting enzyme, PEGylated human arginase I—BCT-100 (NCT03455140), is also undergoing phase I and II clinical trials for the treatment of acute lymphoblastic leukemia [23]. It also has great potential for treating lung cancers such as malignant pleural mesothelioma [24] and SCLC [25]. Although ASS-positive cancer cells are sensitive to BCT-100, its random and multiple 5 kDa PEG conjugation [8] also led to heterogeneity and batch to batch variations.

**Figure 1:**
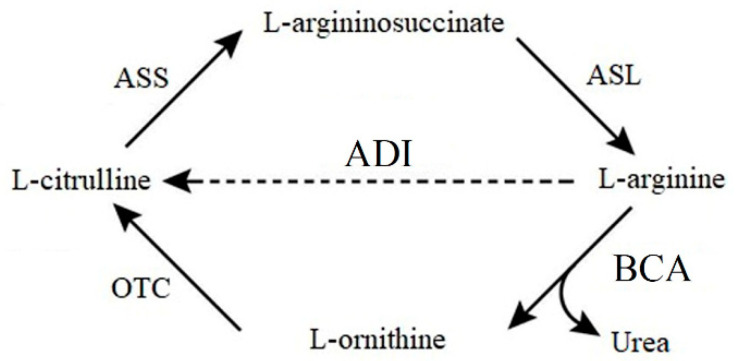
Urea cycle. Absence of ornithine transcarbamylase (OTC) or argininosuccinate synthetase (ASS) renders the cell incapable of synthesizing L-arginine from L-ornithine or L-citrulline, resulting in sensitivity to *Bacillus caldovelox* arginase (BCA) treatment. OTC-negative but ASS-positive cancer cells are resistant to arginine deiminase (ADI) treatment by recycling citrulline to arginine for survival. ASL: Argininosuccinate lyase.

In this study, we attempted to circumvent the current problems by engineering an extremely thermostable arginine-depleting enzyme, *Bacillus caldovelox* arginase (BCA), which is also a bacterial hexameric enzyme [26]. Our data showed that it displayed a high production yield in the soluble fraction in high cell-density fermentation with a simple purification method (74 °C heat treatment: purity > ~90%). Unlike ADI, BCA catalyzes the conversion of L-Arg into L-ornithine instead of citrulline, showing that BCA is capable of killing a broad spectrum of cancer cell types compared to ADI (Figure 1). We have successfully constructed a mono-PEGylated BCA mutant (BCA-M-PEG20) by conjugating one 20 kDa PEG-maleimide which is a stable linkage by double bond addition [17] to a cysteine residue on the protein surface. BCA-M-PEG20 exhibited excellent pharmacodynamic and pharmacokinetic properties in vivo in comparison to the native BCA-M protein. BCA-M-PEG20 caused significant in vitro and in vivo anti-tumor effects in lung cancer cell lines. Moreover, BCA-M-PEG20 did not cause any significant toxicity in terms of hematological values, organ weights and clinical biochemical findings. Our data suggested that BCA-M-PEG20 is a promising candidate for treating lung cancer.

## 2. Results

### 2.1. Optimizing the Feeding Strategy for High Yield of BCA-M Production

**Figure 2:**
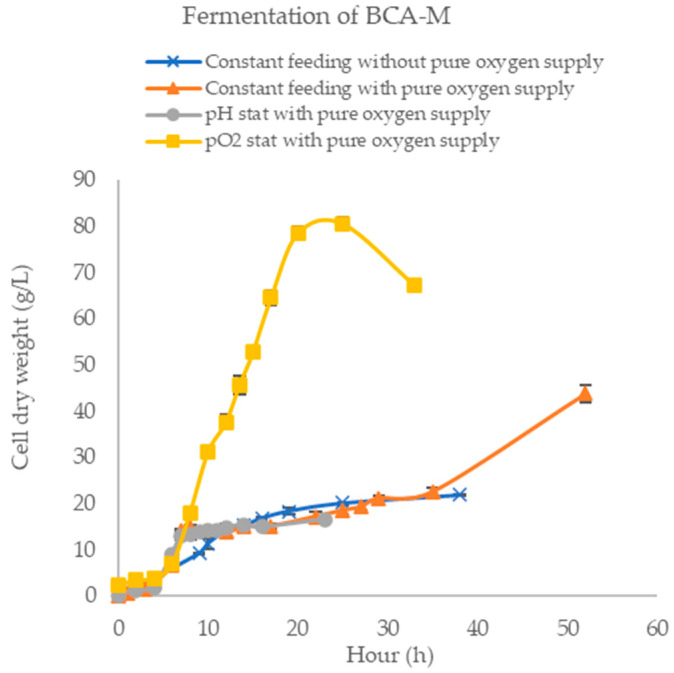
Fermentation optimization in terms of cell dry weight. Growth profile of *Bacillus caldovelox* arginase mutant (BCA-M) fermentation using constant feeding without pure oxygen supply, constant feeding with pure oxygen supply, pH stat with pure oxygen supply and pO2 stat with pure oxygen supply.

BCA-M was produced in fed-batch fermentation with different feeding strategies, as shown in Figure 2. The cell growth was limited in fed-batch fermentation without pure oxygen supply and it entered the stationary phase with a maximum average cell dry weight (CDW) of 21.8 g/L culture at 38 h. Thereby, pure oxygen supply to fermentation was performed. In this process, 60% pure oxygen-to-air ratio was applied to sustain the dissolved oxygen level at above 20% for efficient cell growth, which resulted in a high average CDW of 43.7 g/L. To further enhance cell growth, computerized feeding strategies, pH stat and pO_2_ stat, were investigated. From the growth curve studies using pH stat, the highest average CDW was 16.5 g/L in 29 h fermentation. By using the pO_2_ stat feeding system and the supply of pure oxygen, high cell density culture was achieved with an average CDW of 80.6 g/L in 23 h fermentation. Feeding the cells controlled by pO_2_ stat with pure oxygen supply resulted in the highest cell density. Figure 2 and Table 1 show the summary of the highest cell dry weight by the four feeding strategies.

With the optimal fermentation condition for BCA-M expression using the pO_2_ stat feeding strategy, the BCA-M expression profile after isopropyl β-d-1-thiogalactopyranoside (IPTG) induction was studied (Table 2). The BCA-M yield and expression efficiency were increased with the duration of expression extended from 10 to 19.5 h. The optimized BCA-M yield was much higher than that reported from various ADI production [12,27,28,29,30,31,32] (Table 3).

### 2.2. PEGylation of BCA-M

**Figure 3:**
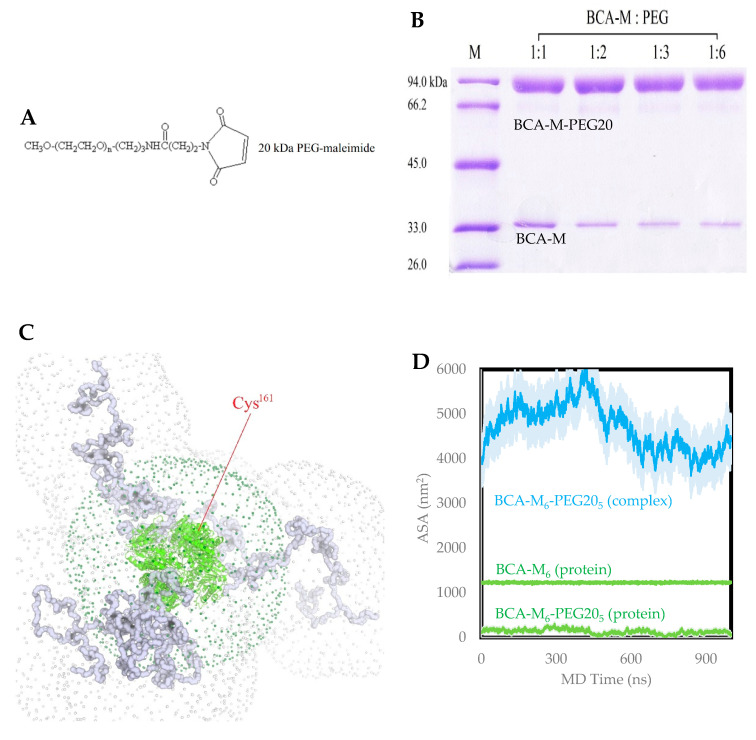
PEGylation optimization and molecular dynamics (MD) study of BCA-M. (**A**) The chemical structure of 20 kDa PEG-maleimide. (**B**) BCA-M was reacted with 1, 2, 3 and 6 molar equivalents of PEG 20 kDa. (**C**) Coarse-grained molecular dynamics snapshot: the BCA-M_6_ is shown as green cartoon, with the PEG20 chains as grey tubes. The grey dotted surface depicts the solvent-accessible surface (SAS) of BCA-M_6_-PEG20_5_ probed with a sphere of 4.9 nm radius. The dark green dotted surface is the SAS of the BCA-M_6_ only. The only Cys^161^ residue that does not have PEG20 attached is indicated. (**D**) The total accessible surface area (ASA) of BCA-M_6_ (middle, green) and BCA-M_6_-PEG20_5_ (cyan) are shown. The protein component of the total ASA of BCA-M_6_-PEG20_5_ is the lower curve (green). The standard deviation throughout MD time was used as error bars.

The chemical structure of 20 kDa PEG-maleimide used for BCA-M conjugation is shown in Figure 3A. The amount of PEG needed for successful PEGylation was evaluated by adding 1 to 6 molar equivalents of 20 kDa PEG (PEG20) to BCA-M. There were no further improvement beyond the BCA-M:PEG ratio of 1:2 (Figure 3B). The modification level achieved was about 85% (estimated by ImageJ (National Institutes of Health, Bethesda, USA)). To investigate the factors influencing the PEGylation efficiency, we performed a long 1 μs coarse-grained molecular dynamics (CGMD) experiment on a model of the BCA-M_6_-PEG20_5_ complex (hexameric BCA-M with 5 protomers each conjugated with one PEG20). Throughout the MD duration, the five PEG20 chains were observed to be able to adopt highly variable coil-like conformations in the solvent volume. The overall effect was that the PEG20 chains did not stay wrapped around the protein, nor formed stable ‘folded’ conformations. Instead, the PEG20 chains tended to form transient residual local secondary structures. Both PEG–PEG associations and PEG–protein interactions were found (Figure 3C). Probed with a 4.9 nm-radius sphere, which represented a compact PEG20 molecule, the hexameric BCA-M_6_ protein had an accessible surface area (ASA) of 1230 ± 14 nm^2^. With five PEG20 chains attached, the total ASA of the complex increased to between 4000 and 6000 nm2, with a mean of 4707 ± 523 nm^2^. However, the protein ASA contribution was reduced to only 134 ± 51 nm^2^ (Figure 3D). The much-reduced ASA could explain why it was difficult to add the sixth PEG20.

### 2.3. Characterizations of BCA, BCA-M and BCA-M-PEG20

**Figure 4:**
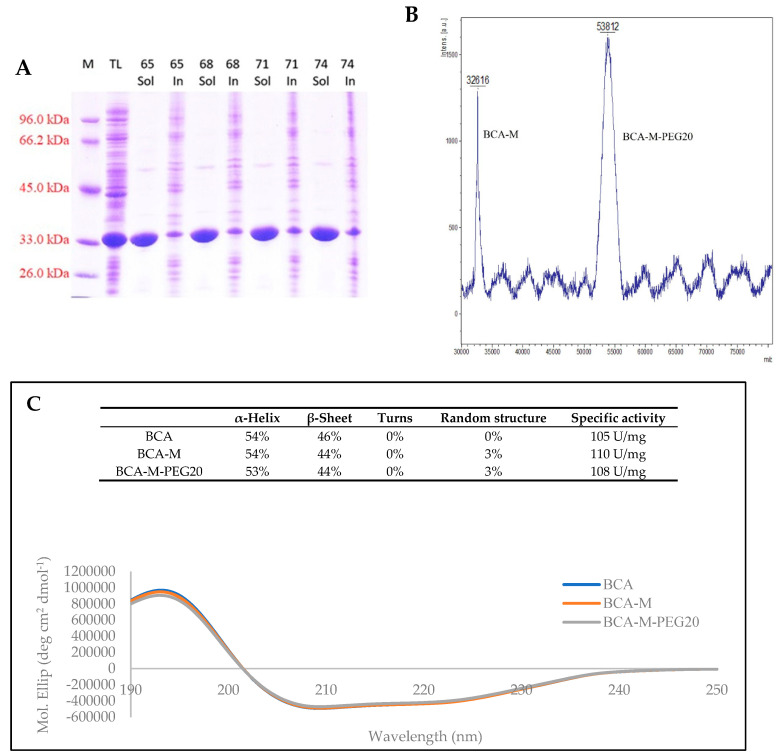
Characterization of BCA, BCA-M and BCA-M-PEG20. (**A**) BCA-M purification by heat treatment, from 65 to 74 °C. M = marker; TL = total lysate; Sol = soluble fraction; In = insoluble fraction. (**B**) MALDI-TOF-MS spectrum of BCA-M-PEG20 (expected molecular weight = 32.45 kDa). (**C**) Circular dichroism (CD) spectra and specific activities of BCA, BCA-M and BCA-M-PEG20.

Being a thermostable enzyme in nature, BCA-M (the S161C mutant) of very high purity was obtained by simple heat treatment at 74 °C (Figure 4A). This feature is very important in industrial production because it significantly reduces the purification cost.

As shown in Figure 4B, the attachment of a single molecule of PEG20 to BCA-M was expected at the position of Cys^161^, as confirmed by mass spectrometry (MS). The calculated molecular weights of BCA-M and BCA-M-PEG20 are 32.6 kDa and 53.8 kDa, respectively.

The circular dichroism (CD) spectra and the measurement of specific activities of BCA, BCA-M and BCA-M-PEG20 showed that there was no significant structural change after the point mutation (S161C) and the attachment of the PEG20 on BCA-M (Figure 4C).

### 2.4. BCA-M-PEG20 Induced Potent Cytotoxicity in Lung Cancer Cell Lines A549, DMS114, NCI-H23 and NCI-H460

**Figure 5:**
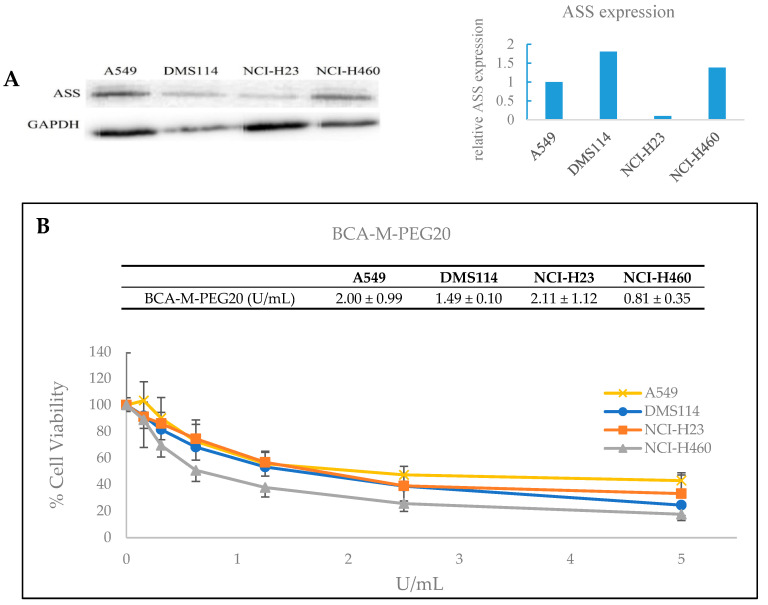
Western blot analysis and growth inhibitory effects of BCA-M-PEG20. (**A**) The protein expression levels of ASS were determined on A549, DMS114, NCI-H23 and NCI-H460 lines. (**B**) Growth inhibitory effects of BCA-M on the four cell lines. IC_50_ values are given and the results are expressed as means ± S.D. Three independent experiments were performed for each cell line.

BCA-M-PEG20 of various concentrations was applied into the culture medium of A549, DMS114, NCI-H23 and NCI-H460 cell lines. All these lung cancer cell lines were ASS-positive, as confirmed by Western blot (Figure 5A). The 50% growth inhibition (IC_50_) values of BCA-M-PEG20 on A549, DMS114, NCI-H23 and NCI-H460 were 2.00 ± 0.99, 1.49 ± 0.10, 2.11 ± 1.12 and 0.81 ± 0.35 U/mL, respectively (Figure 5B).

### 2.5. Pharmacodynamics and Pharmacokinetics Studies of BCA-M and BCA-M-PEG20

**Figure 6:**
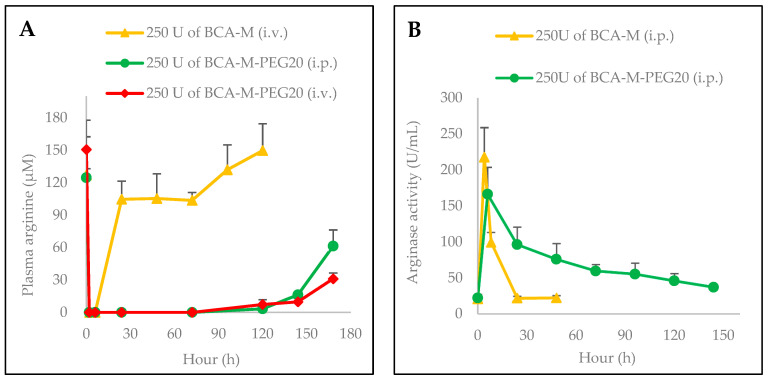
Pharmacodynamics (*n* = 3) and pharmacokinetics (*n* = 5) studies of BCA-M and BCA-M-PEG20 in normal BALB/c mice following a single i.p. and i.v. administration. Plasma was collected from saphenous veins in mice hind legs at the indicated time points. The concentrations of serum arginine (**A**) and arginase activity (**B**) were determined by amino acid analysis and QuantiChrom Urea Assay Kit, respectively. Results are presented as mean ± SEM.

The effects of BCA-M and BCA-M-PEG20 treatment on plasma L-Arg in mice are shown in Figure 6. A single i.p. or i.v. injection of 250 U/mouse of BCA-M-PEG20 could sustain a low level of serum L-Arg for up to 168 h (Figure 6A), while a single injection of BCA-M at 250 U/mouse could only maintain low level of L-Arg for 6 h (Figure 6A). The drug elimination half-lives of BCA-M-PEG20 and BCA-M were 91.4 and 6.4 h, respectively (Figure 6B). BCA-M-PEG20 resulted in much longer serum L-Arg deprivation and better serum stability compared to BCA-M.

### 2.6. Anti-Tumor Activity of BCA-M-PEG20 in the A549 Xenograft Mouse Model

**Figure 7:**
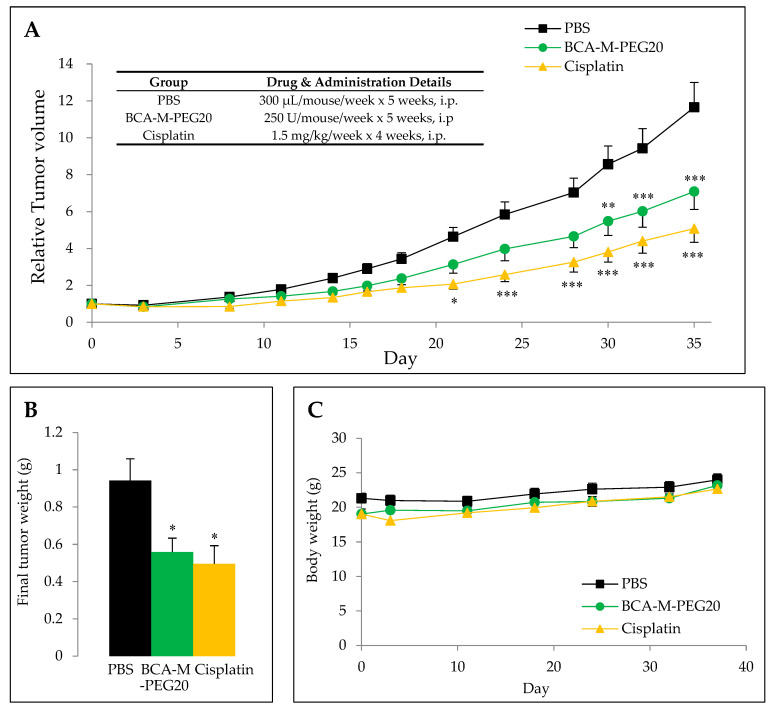
In vivo antitumor activities of BCA-M-PEG20. (**A**,**B**) Tumor growths (relative tumor volume and final tumor weight) were significantly suppressed by BCA-M-PEG20 and cisplatin (*n* = 10). (**C**) Mice body weights were measured regularly. Results are presented as means ± SEM. For relative tumor volume, multiple comparisons were performed using two-ways ANOVA with Bonferroni’s post-tests. For final tumor weight, data were analyzed using Mann–Whitney U-test. * *p* < 0.05, ** *p* < 0.01 and *** *p* < 0.001. All analyses were performed using the Prism software (GraphPad Software Inc, La Jolla, CA, USA).

The in vivo anti-tumor drug efficacies of BCA-M-PEG20 and cisplatin (positive control) on nude mice bearing human A549 xenograft were determined. The drug dose regimen chosen (Figure 7A inset table) was based on the results of pharmacological studies. Progressive tumor growth was observed in the control group, whereas significant tumor suppression was observed in the treatment groups of BCA-M-PEG20 and cisplatin, as shown in Figure 7A,B. There was no observable body weight loss registered during the experiments, as shown in Figure 7C.

### 2.7. Safety Evaluation of BCA-M-PEG20 Treatment in Mice

Regarding the safety of BCA-M-PEG20 administration, the survival of the mice was monitored. No drug-related mortality has ever been recorded for single or repeated weekly administrations of BCA-M-PEG20 via extravascular routes using normal BALB/c mice or nude mice. In this drug safety evaluation using normal BALB/c mice, the results show that the administration of BCA-M-PEG20 did not induce any significant changes in the final weights of the bodies or organs of mice, including the brain, heart, lung, liver and spleen. Complete blood count profiles of the mice treated with BCA-M-PEG20 or PBS vehicle control showed no significant changes in white blood cell count (WBC), red blood cell count (RBC), hemoglobin content (Hb), hematocrit (HCT), mean corpuscular volume (MCV), mean corpuscular hemoglobin (MCH), corpuscular hemoglobin concentration (MCHC), red blood cell distribution (RDW), platelet count (PLT) and mean platelet volume (MPV). Furthermore, clinical blood biochemistry tests also indicated that BCA-M-PEG20 administration did not induce any significant changes in the levels of alanine aminotransferase (ALT) activity, albumin (ALB), albumin (ALB), total plasma protein (TP), urea nitrogen in blood (BUN), creatinine, total cholesterol (TCH), and triglycerides (TG) (Table 4).

## 3. Discussion

Previous research has demonstrated that random PEGylation applied on ADI (ADI-PEG20) could sustain a low level of serum L-Arg from <24 h to ~192 h (ADI-PEG20) [16]. Although it is a promising anti-tumor agent for treating several types of cancers [6,7,21,33] and it is undergoing phases II and III clinical trials [10], it has three disadvantages. Kaur et al. [28], Kim et al. [29], Noh et al. [27], Park et al. [12], Patil et al. [30], Takaku et al. [31] and Weickmann et al. [32] showed that the production yields of ADI were low (0.6–20 mg/L), mainly due to the formation of inclusion bodies and its complicated purification processes [15]. In addition, Western blot results showed that the four lung cancer cell lines were ASS-positive, suggesting that they were resistant to ADI treatment [5,7,8,11,18,19,20,21,22]. Furthermore, random PEGylation produces a mixture of products with batch-to-batch variations.

*Bacillus caldovelox* is a thermophilic bacterium isolated in Yellowstone National Park [34]. Its proteins have high thermostability which offers great benefits in their industrial production and can significantly reduce the cost of protein purification since a simple heat treatment can be applied [35]. Our results show that BCA-M of high purity (>90% purity as shown in Figure 4A) was easily obtained by a 74 °C treatment. In addition, high production yield of BCA-M can be achieved in *E. coli* using the fed-batch fermentation. To maximize the production yield of BCA-M, high cell density culture is one of the fermentation strategies. Gaseous exchange is one of the major limiting factors in high cell growth. Increments in stir rate and airflow are standard methods used to enhance gaseous exchange during fermentation. These methods were evaluated in constant feeding without pure oxygen supply which resulted in low cell mass (21.8 g/L). The cell mass was significantly increased by pure oxygen supply in constant feeding (43.7 g/L) but not in pH stat (16.3 g/L). It was noted that the medium feeding rate of pH stat was too low to provide a large enough carbon source to support the exponential growth of *E. coli* under pure oxygen supply. To this end, pO_2_ stat was studied. It was found that the use of pO_2_ stat could enhance cell growth significantly (88.7 g/L). The optimized production yield of BCA-M was 1625 mg/L after 19.5 h IPTG induction, which was more than 80-fold higher than that of ADI. The high production yield of BCA-M in high cell density fermentation combined with simple purification could significantly reduce the drug production cost.

An MTT-based in vitro anti-cancer efficacy assay showed that BCA-M-PEG20 has a remarkable cytotoxic effect on various ASS-positive lung cancer cell lines, namely, A549, DMS114, NCI-H23 and NCI-H460. These cancer cell lines would probably be resistant to ADI-PEG20 due to their significant ASS expression [5,7,8,11,18,19,20,21,22], which allow the catalytic product of ADI-PEG20, citrulline, to be recycled to form arginine via the urea cycle.

The mono-PEGylation strategy ensures drug homogeneity and assures the quality of the drug [36,37,38,39,40]. BCA-M was genetically modified to replace a serine with a cysteine residue (Ser^161^ → Cys^161^) that would subsequently be used for the coupling of a 20 kDa PEG-maleimide. This mono-PEGylation method retained the secondary structure of the protein and there was no loss of enzymatic activity (Figure 4C). The conjugation of BCA-M with 20 kDa PEG was highly efficient, yielding at least 85% BCA-M-PEG20 (Figure 3B). A similar strategy for mono-PEGylation of human arginase I was performed by Stone et al.; however, only about 50% of target protein was found to be PEGylated [41].

Based on the molecular dynamics simulation results, the five attached PEG20 molecules located on the BCA-M_6_ hexamer were shielding the last Cys^161^ residue for further addition. After the attachment of the five PEG20 molecules, the accessible surface area of the BCA-M_6_ protein significantly decreased from 1230 to 134 nm^2^ (Figure 3), meaning that approximately 90% of the protein surface was shielded. In reality, a PEG20 molecule is hardly spherical, but treating it as such did give us a semi-quantitative explanation for the substantially reduced probability of its gaining access and reacting successfully.

BCA-M-PEG20 showed remarkable enhancement in drug circulating and elimination half-lives when compared to the BCA-M. Our results revealed that the injection of 250 U/mouse of BCA-M-PEG20 provided long-lasting serum arginine depletion for 168 h, as compared with only 6 h with the BCA-M (Figure 6A). In addition, the results of the pharmacodynamic study of BCA-M-PEG20 are similar to those of ADI-PEG20 [16]. Long serum L-Arg depletion reduces the frequency of drug administration by injections and thus alleviates the suffering of patients. Long-lasting depletion also significantly reduces the chance of anti-drug antibody development. Regarding drug pharmacokinetic, a single administration of BCA-M-PEG20 at 250 U/mouse i.p. showed a ~14-fold longer drug elimination half-life of 91.4 h in comparison to the 6.4 h of the BCA-M (Figure 6B). These significant improvements might be attributed to a strong shielding effect of the 20 kDa PEG modification on BCA-M that protected the protein surface against proteases and prevented the protein from renal clearance.

For in vivo anti-tumor efficacy tests of BCA-M-PEG20 and cisplatin on nude mice bearing A549 tumor xenografts, weekly administration of 250 U/mouse of BCA-M-PEG20 and weekly administration of 1.5 mg of cisplatin exhibited ~41% and ~47% tumor suppression, respectively. These results show that BCA-M-PEG20 is a potential candidate as good as cisplatin for treating ASS-positive lung cancers.

Toxicity and strong side effects of chemotherapies have always been a problem and limiting factor for most of the conventional anticancer drugs such as cisplatin, doxorubicin, and 5-fluorouracil. The urge for the development of effective and safe alternatives has so far been the focus of anticancer drug research worldwide. Drug safety evaluation using normal BALB/c mice in this study showed that BCA-M-PEG20 administration for at least 6 weeks was very well tolerated (Table 4). There were no significant changes in the final body weight, organ weight and hematological profile in the normal BALB/c mice administered with BCA-M-PEG20 compared with those injected with PBS control vehicle. Furthermore, biochemical markers such as ALT for liver function, creatinine for kidney function and total cholesterol and triglycerides for cardiovascular risk indicated no toxicity towards the liver function, kidney function and cardiovascular system after administration of BCA-M-PEG20 in the normal BALB/c mice.

## 4. Materials and Methods 

### 4.1. Materials

Materials not specified here were obtained from Sigma Chemical Company (St. Louis, MO, USA). Cancer cell culture kit with MTT (3-(4,5-dimethylthiazol-2-yl)-2,5-diphenyltetrazolium bromide) reagent, as well as all cell culture media and sera, were purchased from Invitrogen Life Technologies (San Diego, CA, USA). Meanwhile, 20 kDa PEG-maleimide (Mw 20,000) was obtained from NOF America Corporation (White Plains, NY, USA). Expression plasmid pET3a was obtained from Invitrogen (San Diego, CA, USA). Human lung cancer cell lines A549, DMS114, NCI-H23 and NCI-H460 were obtained from American Type Culture Collection (ATCC; Manassas, VA, USA). Cell lines were maintained in RPMI-1640 medium supplemented with 10% fetal bovine serum and 100 units/mL of penicillin/streptomycin (Gibco, UK).

### 4.2. Molecular Cloning Techniques

The plasmid pET3a/BCA was used as a template for site-directed mutagenesis according to the instruction of the QuikChange site-directed mutagenesis kit (Strategene, CA, USA). The codon for Ser^161^ residue was mutated to the codon for Cys^161^ using the following mutagenic primers. The mutated BCA was named as BCA-M. 

Forward primer S161C 5′ CAAATCGGCGGATAC***T***GCCCCAAAATCAAGC 3′; 

Reverse primer S161C 5′ GCTTGATTTTGGGGC***A***GTATCCGCCGATTTG 3′.

### 4.3. Fermentation of BCA-M

Fed-batch fermentations were carried out in a 2 L bioreactor with 1.25 L modified 2xTY medium (40 g/L glycerol + 2 g/L MgSO_4_ + 3 g/L K_2_HPO_4_ + 5 g/L KH_2_PO_4_ + 1.7 g/L (NH4)_2_SO_4_ + 3 mL trace element) and 500 mL enriched feeding medium (400 g/L glycerol + 30 g/L (NH_4_)_2_SO_4_ + 7.2 g/L MgSO_4_). To prepare the seed inoculums for fed-batch fermentation, 4 mL of cultured medium from a single colony were transferred into 150 mL of seed medium. Then, all of the seed medium was transferred into 1.25 L of fermentation medium for high cell density fermentation. Feeding was started when the initial carbon source was used up, as indicated by a significantly rise in PO_2_ level. Four feeding strategies were carried out for high cell density fermentation, constant feeding without pure oxygen supply (feeding and IPTG addition started after 9 and 12 h culturing, respectively), constant feeding with pure oxygen supply (feeding and IPTG addition started after 12 h culturing), pH stat and pO_2_ stat. For pH stat, a computer-controlled feeding system based on the monitoring of the pH of the fermentation medium to determine the availability of carbon source. Once the pH rose due to carbon source depletion, a predetermined amount of feeding solution was fed into the fermenter (feeding and IPTG addition started after 12 h culturing). For pO_2_, a computer-controlled feeding system based on the relationship of carbon source availability and pO_2_ value was used. When the carbon source was depleted, the pO_2_ value rose and a predetermined amount of feeding solution was fed into the fermenter (feeding and IPTG addition started after 8 and 13.5 h culturing, respectively). Additionally, 0.5 mM isopropyl β-d-1-thiogalactopyranoside (IPTG) was introduced into the fermentation broth for recombinant *Bacillus caldovelox* arginase mutant (BCA-M) expression.

### 4.4. Preparation of BCA-M-PEG20

Recombinant *Bacillus caldovelox* arginase mutant (BCA-M) was obtained via over-expression of the BCA-M enzyme in *E. coli* and purification using heat treatment and nickel chelation affinity chromatography [42]. One to six molar equivalents of 20 kDa PEG were added into purified the BCA-M (3 mg/mL) and incubated at 4 °C for overnight. The specific activities of BCA, BCA-M and BCA-M-PEG20 were ~105, 110 and 108 Units/mg (U/mg), respectively.

### 4.5. Specific Activity

The chromophore compound was detected at a wavelength of A_530_ nm in the presence of diacetyl monoxime, thiosemicarbazide, urea and Fe^3+^ under high temperature [43]. L-Arg solutions (200 μL and 20 mM) were incubated at 37 °C using a heat block. Reactions were started by adding 5 μL of arginase (0.02 mg/mL) and stopped with 15 μL of 80% trichloroacetic acid. The reaction time was 30 s. A coloring reagent was prepared by 1 volume of a mixture of 80 mM diacetyl monoxime and 2.0 mM thiosemicarbazide and 3 volumes of a mixture of 3 M H_3_PO_4_, 6 M H_2_SO_4_, 2 mM FeCl_3_. Eight hundred microliters of the coloring reagent was added to each reaction, and then the reaction mixtures were incubated at 100 °C for 10 min followed by cooling down for 5 min at room temperature. A_530_ nm was determined using ultraviolet-visible spectroscopy (Spectronic 20 Genesys Spectrometer). One international unit of arginase is defined as the amount of enzyme that can produce 1 μmol urea/min at 37 °C, pH 7.4.

### 4.6. CD Spectroscopic Analysis

The secondary structure content of the enzyme was determined using the Jasco J-810 spectropolarimeter (Great Dunmow, UK). For secondary structure, samples were monitored in the far-UV region (190–250 nm) and CD spectra were obtained from an average of 3 scans.

### 4.7. The Degree of PEGylation of BCA-M-PEG20 Determination

Before the analysis, enzymes were desalted by a C4 Ziptip according to the manufacturer’s instruction (Millipore,Sigma-Aldrich, Burlington, Mass, USA). The desalted samples were mixed with a sinapinic acid matrix solution, 70% acetonitrile and 0.1% trifluoroacetic acid, and then analyzed using MALDI-TOF-MS with a 355 nm Nd:YAG laser source. The mass spectrometer was operated in positive linear mode during data acquisition.

### 4.8. Molecular Dynamics Simulation

The hexametric BCA complex (BCA_6_) from the crystal structure with PDB ID: 2CEV was used (two crystallographically related A-, B-, C-chain trimers). The protein model was transformed into a coarse-grained (CG) representation by the martinize.py script [44]. A 450-monomer PEG chain (approximately 20 kDa) was ‘grown’ and linked to the side-chain bead of residue S161C (BCA-M) in five of the six BCA-M protomers using the POLYply. Script [45]. The BCA-M_6_-PEG20_5_ molecular model was subjected to energy minimization in vacuo. Subsequently, the model was immersed in a cubic solvent box of sides of 45 nm, which was large enough to allow the PEG chains to explore extended conformations without restriction. The system was solvated with martini water beads, and with Na^+^ (60) added to neutralize the charges on the protein. The complex model was subjected to steepest-slope energy minimization. Next, the system was equilibrated for 50 ns (0.02 ps timesteps) in an NPT ensemble (keeping the number of particles, pressure, and temperature constant). The production run consisted of 1 μs of restrained MD. The simulation was performed with Gromacs 2018.4 [46] using the martini v2.2 forcefield [44] with PEG implementation [45]. Throughout all stages, all protein beads were restrained with a force constant of 1000 kJ mol^−1^ nm^−2^.

The MD results were visualized with VMD (https://www.ks.uiuc.edu/Research/vmd/) and PyMOL (https://pymol.org). The Gromacs tool ‘gmx sasa’ was used to calculate solvent accessible surface areas. The hydrodynamic radius of a PEG20 molecule was estimated to be 4.9 nm [47]. We probed the accessible surface areas of the BCA-M_6_ protein on its own, as well as with penta-PEGylation.

### 4.9. MTT-Based In Vitro Anti-Cancer Efficacy Assay

Non-small cell lung cancer of adenocarcinoma (A549 and NCI-H23) and non-small cell lung cancer of large cell carcinoma (NCI-H460) and small cell lung cancer (DMS114) were used in the in vitro anti-cancer assay. Almost all lung cancer cell types were covered. Three thousand cancer cells per well were seeded in a 96-well plate. After treatment with BCA-M-PEG20 for 72 h, quantitative cell proliferation assays were performed by addition and incubation for 4 h with 10 µL per well of 5 mg/mL MTT reagent [3-(4,5-dimethylthiazol-2-yl)-2,5-diphenyltetrazolium bromide], followed by a dissolution of the formazan using 10% SDS and absorbance reading at 540 nm using a CLARIOstar^®^ plate reader (BMG LABTECH, Weston, FL, USA). The absorbance from untreated cells was considered 100% cell viability and drug dosages required to produce 50% growth inhibition (IC_50_) were calculated using the Prism software (GraphPad Software Inc.) with the non-linear regression sigmoidal dose–response curve model. Three independent experiments were performed in at least triplicate.

### 4.10. Western Blot Analysis

Lung cancer cells were centrifuged at 1500 rpm for 5 min at 4 °C and washed with cold phosphate-buffered saline and kept on ice in RIPA lysis buffer (Thermo Fisher Scientific, Rockford, IL, USA). Lysates were centrifuged at 12,000 rpm for 10 min at 4 °C and the supernatants were kept for Western blot analysis. Equal amounts of sample were applied to an SDS-PAGE gel, then the gel was transferred into a polyvinylidene fluoride (PVDF) membrane. The transferred membrane was blocked with 10% non-fat milk, then incubated with the primary antibodies, rabbit anti-GAPDH antibody (1:2500, Abcam, USA) and rabbit anti-ASS1 antibody (1:20,000, Abcam, USA). After the incubation, the membrane was incubated with HRP-conjugated goat anti-rabbit IgG (1:5000, Abcam, USA); then, signals were detected by a chemiluminescent detection kit (Pierce, Rockford, IL, USA). The relative ASS expression level was estimated by ImageJ.

### 4.11. Pharmacodynamic and Pharmacokinetic Studies

For pharmacodynamic studies, normal BALB/c mice, *n* = 3 and 6–8 weeks old, were randomly assigned into groups. A single i.v. or i.p. injection of 250 U/mouse of BCA-M or BCA-M-PEG20 were administered on day 0. Blood samples were collected from the saphenous vein in the hind leg before (0 h) and after drug administration at the time points of 2, 6, 24, 48, 72, 96, 120, 144 and 168 h. Collected samples were mixed with 10% sulfosalicylic acid for protein precipitation. The supernatant was collected and mixed with an equal volume of lithium citrate loading buffer (Biochrom, Cambourne, UK), then applied to the amino acid analyzer (model L-8800, Hitachi) for plasma arginine level determination.

For pharmacokinetic studies, normal BALB/c mice, *n* = 5 and 6–8 weeks old, were randomly assigned into different groups with single i.p. injections of 250 U/mouse of BCA-M or BCA-M-PEG20. Plasma samples collected before and after drug administration were purified from urea content using microcon^®^ centrifugal devices (Millipore). Then, plasma drug activities were determined using the QuantiChrom urea assay kit (Bioassay systems) and used for the determination of drug elimination half-lives (t_1/2_) and pharmacokinetic descriptive statistics using two compartment models.

### 4.12. In Vivo Anti-Tumor Efficacy Test of BCA-M-PEG20

The athymic nude mice, *n* = 10, were purchased from The University of Hong Kong and housed under pathogen-free conditions at The Hong Kong Polytechnic University (Centralised Animal Facility, CAF) using the IVC system according to institutional guidelines and provided with drinking water and standard mouse chow ad libitum. A549 cells (1 × 10^7^) were subcutaneously inoculated to the mice with the supplement of matrigel for the establishment of the A549 xenograft model. The mice were randomly divided into the control (0.9% normal saline) and treatment (BCA-M-PEG20 and cisplatin) groups. The dosage of BCA-M-PEG20 used was based on the pharmacodynamic studies, whereas the dosage of cisplatin used was based on a research paper [48]. The solid tumors were monitored regularly by digital caliper and tumor volumes were calculated with the formula 1/2 × L × W2, where L and W are the length of the longest dimension and its perpendicular axis of the tumor, respectively. Relative tumor volumes of each mouse were also calculated with reference to day 0 of the same mice. The mice were euthanized by cervical dislocation at the end of the experiment. Then, the final tumor and body weights were recorded and the % final tumor-to-body weight was calculated with the formula 100% × final tumor weight/final body weight. Body weights of the mice were also recorded regularly throughout the experiment.

### 4.13. Assessment of Changes in Body and Organ Weight, Complete Blood Count and Blood Chemistry during Arginine Depletion in Mice

Normal BALB/c mice were obtained from the Central Animal Facilities (CAF) of the Polytechnic University of Hong Kong. The mice were housed according to institutional guidelines and provided with drinking water and standard mouse chow ad libitum. The mice were randomly separated into 2 groups (*n* = 4) and administered i.p. with BCA-M-PEG20 (250 U/mouse) or PBS (control vehicle) once per week for 6 weeks. At the end of the experiment, the final body and organ weights of the mice were determined, while blood samples were collected for complete blood count and blood chemistry tests.

## 5. Conclusions

In this study, BCA-M-PEG20 was demonstrated to be a promising anticancer drug candidate. High cell density fermentation for large scale production of BCA-M was successfully achieved by pO_2_ stat with pure oxygen supply strategy. Being thermostable in nature, the drug offers great benefits for a much simpler industrial production at a lower cost. BCA-M was also highly effective over a board spectrum of lung cancer cells regardless of their expression of ASS. In addition, BCA-M-PEG20 showed excellent drug homogeneity, pharmacokinetics, and pharmacodynamics properties. Regarding its antitumor efficacy, BCA-M-PEG20 exhibited excellent cytotoxic effect on A549 human non-small cell lung adenocarcinoma tumor xenografts in vivo. Finally, toxicological evaluation of BCA-M-PEG20 on normal BALB/c mice indicated that the drug is safe and well-tolerated.

## Figures and Tables

**Table 1:** Comparison of the highest cell dry weight and optical density produced by different feeding strategies.

Experimental Trial	Constant Feeding without Pure Oxygen Supply	Constant Feeding with Pure Oxygen Supply	pH Stat with Pure Oxygen Supply	pO_2_ Stat with Pure Oxygen Supply
Pure oxygen supply (Max = 60%)	X	✓	✓	✓
Optical density (OD_600_)	35	90	40	191
Cell dry weight (g/L)	21.8	43.7	16.4	80.6

**Table 2:** BCA-M yield and expression efficiency with different duration of expression in the pO_2_ stat fermentation system.

**Duration of expression after IPTG induction (h)**	10	15	19.5
**BCA-M yield** **(mg-BCA-M/L-culture)**	872	1486	1625

**Table 3:** The comparison of the enzyme yields between BCA-M and ADI.

Recombinant Enzymes	Enzyme Yield (mg/L)	References
BCA-M (from *Bacillus caldovelox* arginase)	1625	This study
ADI (from *Mycoplasma arginine*)	20	[27]
ADI (from *Mycoplasma arginine*)	5	[12]
ADI (from *Mycoplasma arthritides*)	9.29	[32]
ADI (from *Mycoplasma hominis*)	0.43	[31]
ADI (from *Enterococcus faecium*)	12.1	[28]
ADI (from *Lactococcus lactis*)	1.65	[29]
ADI (from *Pseudomonas putida*)	0.6	[30]

**Table 4:** Safety evaluation of BCA-M-PEG20 administration in mice.

	PBS Control	BCA-M-PEG20	*p* Values vs. PBS
**Hematological Values**			
WBC (K/uL)	2.3 ± 1.2	2.6 ± 1.5	0.802
RBC (M/uL)	8.5 ± 0.3	8.6 ± 0.2	0.622
Hb (g/dL)	13.7 ± 0.6	13.6 ± 0.7	0.825
HCT (%)	46.8 ± 2.1	46.3 ± 1.2	0.676
MCV (fL)	54.9 ± 0.6	53.7 ± 0.2	0.006
MCH (pg)	16 ± 0.2	15.7 ± 0.6	0.406
MCHC (g/dL)	29.1 ± 0.3	29.2 ± 1.1	0.837
RDW (%)	15.2 ± 0.5	16.3 ± 0.7	0.060
PLT (K/uL)	1519.0 ± 141.4	1620.0 ± 114.0	0.309
MPV (fL)	19.6 ± 0.9	21.7 ± 1.4	0.038
**Organ Weights**			
Body weight (g)	25.2 ± 3.5	24.9 ± 3.6	0.909
Brain (g)	0.42 ± 0.03	0.43 ± 0.01	0.695
Heart (g)	0.11 ± 0.02	0.12 ± 0.02	0.693
Lung (g)	0.14 ± 0.03	0.15 ± 0.01	0.582
Liver (g)	1.15 ± 0.22	1.15 ± 0.15	1.000
Spleen (g)	0.13 ± 0.03	0.13 ± 0.02	0.879
Kidneys (g)	0.17 ± 0.03	0.16 ± 0.04	0.787
**Clinical Biochemical Findings**			
ALT (U/L)	69.8 ± 24.3	56.8 ± 7.5	0.346
AST (U/L)	101.0 ± 14.0	78.0 ± 7.2	0.026
ALB (g/dL)	2.8 ± 0.3	2.8 ± 0.3	0.907
TP (g/dL)	55 ± 0.4	55 ± 0.3	0.924
BUN (mg/dL)	24.0 ± 1.6	23.3 ± 2.0	0.579
Creatinine (mg/dL)	0.24 ± 0.01	0.24 ± 0.01	0.705
TCH (mg/dL)	100.8 ± 12.0	104.5 ± 4.7	0.582
TG (mg/dL)	73.0 ± 5.8	75.5 ± 9.7	0.674

Notes: Results are expressed as mean ± S.D. and were analyzed with Student’s unpaired *t*-test. WBC, white blood cell count; RBC, red blood cell count; Hb, hemoglobin; HCT, hematocrit; MCV, mean corpuscular volume; MCH, mean corpuscular hemoglobin; MCHC, mean corpuscular hemoglobin concentration; RDW, red cell distribution; PLT, platelet count; MPV, mean platelet volume; ALT, alanine aminotransferase; AST, aspartate aminotransferase; ALB, albumin; TP, total plasma protein; BUN, urea nitrogen in blood; TCH, Total cholesterol; and TG, triglyceride.
